# Comparison of a Restricted and Unrestricted Vegan Diet Plan with a Restricted Omnivorous Diet Plan on Health-Specific Measures

**DOI:** 10.3390/healthcare3030544

**Published:** 2015-07-14

**Authors:** Richard J. Bloomer, Trint A. Gunnels, JohnHenry M. Schriefer

**Affiliations:** Cardiorespiratory/Metabolic Laboratory, The University of Memphis, Memphis, TN 38152, USA; E-Mails: trintagunnels@gmail.com (T.A.G.); jmschrfr@memphis.edu (J.M.S.)

**Keywords:** dietary modification, nutrient intake, lipid panel, veganism, oxidative stress

## Abstract

*Background:* We have previously noted beneficial health outcomes when individuals follow a dietary restriction plan in accordance with the Daniel Fast (DF). This is true whether individuals eliminate all animal products or include small amounts of meat and dairy in their plan. The present study sought to compare anthropometric and biochemical measures of health in individuals following a traditional DF (*i.e.*, restricted vegan) or modified DF (*i.e.*, restricted omnivorous; inclusive of *ad libitum* meat and skim milk consumption), with those following an unrestricted vegan diet plan. *Methods:* 35 subjects (six men; 29 women; 33 ± 2 years; range: 18–67 years) completed a 21-day diet plan. Subjects reported to the lab for pre- (day 1) and post-intervention testing (day 22) in a 10 h fasted state. Blood samples were collected and assayed for complete blood count, metabolic panel, lipid panel, insulin, HOMA-IR, C-reactive protein, and oxidative stress biomarkers (malondialdehyde, advanced oxidation protein products, and nitrate/nitrite). Heart rate and blood pressure were measured and body composition was determined via dual energy X-ray absorptiometry. Subjects’ self-reported compliance, mental and physical health, and satiety in relation to the dietary modification were recorded. *Results:* No interaction effects were noted for our outcome measures (*p* > 0.05). However, subjects in the traditional DF group reported an approximate 10% increase in perceived mental and physical health, with a 25% reduction in malondialdehyde and a 33% reduction in blood insulin. Systolic BP was reduced approximately 7 mmHg in subjects assigned to the traditional DF, with an approximate 5 mmHg reduction in subjects assigned to the modified DF and the unrestricted vegan plan. A small (2 mmHg) reduction in diastolic BP was noted for subjects in both DF groups; a slight increase in diastolic BP was noted for subjects assigned to the unrestricted vegan group. An approximate 20% reduction was noted in total and LDL cholesterol for subjects in the traditional DF group, with an approximate 10% decrease for subjects in the modified DF group. No decrease in total or LDL cholesterol was noted for subjects in the unrestricted vegan group. *Conclusion:* These data indicate that both a traditional or modified DF may improve blood pressure and blood lipids in a clinically meaningful manner if these results are sustained over the long term. A traditional DF also results in a significant reduction in blood insulin and oxidative stress. An unrestricted vegan diet may improve systolic blood pressure, but in the absence of measures to strictly monitor adherence, it does not favorably impact other markers of health measured in the present study.

## 1. Instruction

Restriction of caloric intake appears to improve overall health, with multiple mechanisms of actions proposed [1,2,3]. While the quantity of dietary energy intake appears important with regards to overall health, the type and quality of macronutrients contained within meals is also of great importance [4,5]. Our published work with the restricted vegan-based “Daniel Fast” plan highlights the importance of nutrient composition as pertaining to a variety of health-related parameters [6,7,8,9,10].

The Daniel Fast is a biblically-inspired (Daniel 1:8–14) dietary restriction model that eliminates all animal products—in a similar manner as a vegan diet. It involves *ad libitum* intake of specific foods, with the elimination of others. For example, the plan consists of fruits, vegetables, whole grains, legumes, nuts, seeds, and oil. Although the plan resembles a typical vegan diet, which has been reported to yield health-enhancing properties [11,12], the Daniel Fast is more stringent, in that no processed foods, white flour products, additives, preservatives, sweeteners, flavorings, caffeine, or alcohol are allowed. Despite the stringent nature, our prior studies have noted short-term (three-week) compliance rates that exceed 95% in nearly all individuals, with many subjects reporting feeling much more satiated on this plan as compared to their usual dietary intake, resulting in a significant reduction in kilocalorie intake.

That said, many subjects report the desire for the occasional consumption of meat and dairy, in particular if they were to adopt such a dietary plan long-term. Many subjects also comment on their desire to consume coffee and tea, which may hinder long-term compliance. Based on this observation, we most recently compared a “traditional” vegan-based Daniel Fast with a “modified” Daniel Fast—inclusive of one serving per day of lean meat and one cup per day of skim milk [6], with the option of consuming decaffeinated coffee and tea. The two servings of animal products provided approximately 30 extra grams of protein per day, in addition to a small amount of cholesterol and saturated fat, yet results indicated that subjects responded equally as well to the modified version as compared to the traditional version with regards to the measured outcome variables. These findings lead us to believe that perhaps the amount of protein and cholesterol contained within foods may not be of great importance, but rather, the amount of processed ingredients, simple sugar, hydrogenated fatty acids, and other similar “unhealthy” additives may be of greater concern.

Although vegan diets are generally viewed as healthy alternatives to the usual animal-rich “Western Diet” followed by so many individuals, vegan plans call for no restriction on the type, form, or amount of dietary carbohydrate or fat ingested. While the health benefits of vegan diets are well-established, it may be appropriate to favor unrefined carbohydrates in the overall diet, while minimizing processed foods and simple sugars, as well as saturated and trans fats, additives, and preservatives.

Considering our prior experience with the Daniel Fast, coupled with our knowledge of vegan diet plans, in the present study we compared three diet plans: a traditional Daniel Fast devoid of animal products [restricted vegan], a Daniel Fast plus *ad libitum* animal products (restricted omnivorous), and an unrestricted vegan diet for a period of 21 days on selected biochemical and anthropometric markers of health. We hypothesized that the all plans would yield improvements in the selected outcome measures, with the Daniel Fast plans proving more beneficial than the traditional unrestricted vegan plan for certain variables (e.g., blood lipids, blood insulin, oxidative stress biomarkers).

## 2. Materials and Methods

### 2.1. Subjects and Screening

Thirty-six subjects (six men; 30 women) were enrolled in this study, with an average age of 33 ± 2 years and a range of 18–67 years. Subjects were required to be non-smokers and within 18–70 years of age. The body mass index (BMI) of subjects was not restricted. Therefore, the BMI range was 19–45 kg∙m^−2^. Our prior studies included sample sizes similar to what was included in the present work; however, no power analysis was performed to determine the present sample size. Prior to participation, each subject completed a health history, medication and dietary supplement usage, and physical activity questionnaire. The work was approved by the University Institutional Review Board for Human Subjects Research (#2329) and subjects provided written informed consent.

During the first laboratory visit, subjects completed all paperwork and were provided with their group assignment. Subjects were allocated to one of the three groups using a randomized method (e.g., numbering system), with alternation between the three diet groups upon subject screening. Once 12 individuals were assigned to a group, the randomization continued using only the final two groups. Subjects were provided with instructions regarding their assigned diet plan and were provided with food logs for dietary recording. Subjects returned to the lab within one to two weeks to have their baseline assessments performed and to begin the 21-day diet plan. All outcome measures indicated below were measured before (baseline: day 1) and after the diet plan (day 22). Subject data were collected in the morning hours (e.g., 6:00–10:00 am) in a 10 h fasted state.

### 2.2. Diet Plans (Traditional Daniel Fast, Modified Daniel Fast, Unrestricted Vegan Diet)

Following the initial week of dietary recording and all baseline assessments, subjects began their diet plan assignment and followed the plan for 21 days. This timeframe coincided with our prior studies of the Daniel Fast. Subjects assigned to all three groups were informed that they could consume as much food as desired—no restrictions were placed on the quantity of food or beverage consumed. However, restrictions were placed on the type of food and beverage consumed. Specifically, subjects assigned to both Daniel Fast groups were informed that they must eliminate all processed foods, white flour products, additives, preservatives, sweeteners, flavorings, caffeine, and alcohol. Subjects in the traditional Daniel Fast group were also required to eliminate all animal products. On the other hand, subjects assigned to the modified Daniel Fast plan were allowed to eat as much lean meat (fish, chicken, turkey, or red meat) and to drink as much skim milk as desired. Subjects assigned to the unrestricted vegan plan were not allowed to consume any animal products (e.g., red meat, poultry, fish, eggs, cheese, milk, *etc.*). No other restrictions were placed on subjects assigned to the unrestricted vegan diet plan. Subjects were contacted by research assistants frequently (e.g., every three to four days) throughout the study period via email, phone, or text message to remind them of the dietary guidelines and to offer encouragement regarding their adherence to the prescribed plan. Research assistants were also available daily to answer any questions that subjects had concerning food choices.

### 2.3. Outcome Measures and Hypotheses

As in our prior work with the Daniel Fast, we included a wide variety of outcome measures. Of most importance were body mass, blood pressure, and selected biochemical measures such as insulin, C-reactive protein, and blood cholesterol. Based on prior work, we hypothesized that improvement would be observed for all outcome measures in all three diet groups, with potential for greater improvement noted for the two Daniel Fast plans due to the restriction of all processed foods.

### 2.4. Anthropometric Measures, Heart Rate and Blood Pressure

After arriving in the lab, subjects were asked to use the restroom and to empty their bladder. Women were required to perform a urine pregnancy test prior to having the dual energy X-ray absorptiometry (DXA) scan performed (Hologic QDR-4500W; using a four-minute fan array with both total and trunk specific body fat being determined). Height and weight was measured, and body mass index was calculated. In addition, waist and hip circumference measures were obtained using a tension-regulated tape measure.

For the measure of heart rate and blood pressure, subjects were asked to sit in a chair with a cuff placed on their left arm, while resting for 10 min. A 60 s palpation was used to measure heart rate and blood pressure was measured via auscultation. Duplicate measurements were obtained for both heart rate and blood pressure and the average was used in data analysis.

### 2.5. Blood Collection and Biochemical Variables

Blood samples were taken from subjects’ forearm vein. Samples were processed to obtain plasma/serum. Aliquots to be used for the analysis of the lipid specific oxidation biomarker, malondialdehyde (MDA; Northwest Life Science Specialties; Vancouver, WA), were separated and stored at −70 degrees Celsius until analyzed. Aliquots for the protein oxidation marker known as advanced oxidation protein products (AOPP; using reagents purchased from Cell Biolabs; San Diego, CA, USA) were also stored, as were aliquots for the nitric oxide marker known as nitrate/nitrite (NOx; using reagents purchased from Cayman Chemical; Ann Arbor, Michigan). Samples were later thawed and assayed in duplicate. Both MDA and AOPP are commonly used biomarkers of oxidative stress. Since oxidative stress is implicated in the pathogenesis of human disease and the degree of oxidative stress appears to be related to dietary intake (e.g., saturated fat and simple sugar content), inclusion of these variables was important in this work.

Remaining assays were performed with 24 h of sample collection. Complete blood count, comprehensive metabolic panel, and lipid panel were analyzed using automated procedures. Insulin was determined using an immuno-chemiluminescent assay procedure (Roche Modular E170, Roche Diagnostics, Indianapolis, IN, USA). The homeostasis model assessment (HOMA-IR) was used as an index of insulin resistance. This was calculated using the following equation: (fasting glucose (mg∙dL^−1^) × fasting insulin (µU∙mL^−1^))/405. C-reactive protein was determined using a high-sensitivity, particle-enhanced turbidimetric immunoassay (Roche Integra 800, Roche Diagnostics, Indianapolis, IN, USA).

### 2.6. Dietary Records and Physical Activity

All subjects were instructed to maintain their usual diet until they began the assigned diet plan. They were also asked to record all food and drink consumed during the seven days before starting the assigned plan. They were asked to do the same during the final seven days of the fast. Records were reviewed with subjects upon receipt and were analyzed by using Food Processor SQL (ESHA Research, Salem, OR, USA). Subjects were instructed to maintain their usual physical activity habits throughout the study period and to avoid alcohol consumption and strenuous exercise during the two days preceding the assessment days.

### 2.7. Compliance, Subjective Feelings, and Satiety

On a scale of 0–100 (0 = complete non-compliance, 100 = complete compliance), subjects rated their overall compliance to the assigned diet plan, in regard to food choices. Using a scale of 0–10 (0 = as low as possible, 10 = as high as possible), subjects rated their overall “mental outlook and mood,” their “physical health and vitality,” and their “level of satiety” both before and while following the assigned diet plan.

### 2.8. Statistical Analysis

Data were analyzed using a 3 (group) × 2 (time) analysis of variance. Tukey post-hoc testing and contrasts was performed as needed. For comparisons reported in the Results section, we note whether we are referring to a group effect (comparing one group to another while collapsing across time) or a time effect (comparing pre and post-intervention while collapsing groups). Any comparisons involving specific groups at specific times involve post hoc testing. Analyses were performed using JMP statistical software (version 4.0.3, SAS Institute, Cary, NC, USA). Statistical significance was set at *p* ≤ 0.05. The data are presented as mean ± SEM.

## 3. Results

Of the 36 subjects that were initially enrolled in the study, one subject assigned to the unrestricted vegan plan did not complete the study due to personal reasons. Therefore, data are only available for 35 subjects (*n* = 12 for both traditional and modified DF; *n* = 11 for unrestricted vegan). Blood was not available for one subject assigned to the modified DF plan. CRP values for one subject assigned to the modified DF plan were significantly elevated and considered to be outliers; they were not included in the analysis. Finally, blood to be used for analysis of oxidative stress biomarkers was not available for all subjects. Specifically, for MDA, blood was available for a total of 11 subjects in the traditional DF, six subjects in the modified DF, and 11 subjects in the unrestricted vegan; For AOPP, blood was available for a total of 10 subjects in the traditional DF, six subjects in the modified DF, and eight subjects in the unrestricted vegan; for NOx, blood was available for a total of 11 subjects in the traditional DF, seven subjects in the modified DF, and 10 subjects in the unrestricted vegan. Dietary data was not available for two subjects in the traditional DF and one subject in the unrestricted vegan plan.

### 3.1. Compliance, Mental Outlook/Mood, Physical Health/Vitality, and Satiety

Differences in self-reported compliance to the prescribed dietary plan could not be detected by the investigators (*p* = 0.47) and was as follows: 96.5 ± 1.0 for the traditional DF, 93.4 ± 2.2 for the modified DF, and 94.1 ± 2.2 for the unrestricted vegan plan. Although subjects assigned to the traditional DF group reported an approximate 10% increase in perceived mental and physical health, no group, time, or interaction effects of statistical significance were detected (*p* > 0.05). A time effect was noted for satiety, with values decreasing across time (*p* = 0.005). Data are presented in Table 1.

**Table 1:** Subject compliance, subjective feelings, and satiety before and after a 21-day period of dietary modification.

Variable	Traditional DF Pre	Traditional DF Post	Modified DF Pre	Modified DF Post	Unrestricted Vegan Pre	Unrestricted Vegan Post
Compliance (%)	NA	96.5 ± 1.0	NA	93.4 ± 2.2	NA	94.1 ± 2.2
Mental Health (1–10)	7.9 ± 0.5	8.7 ± 0.4	8.2 ± 0.5	7.8 ± 0.5	8.5 ± 0.5	8.2 ± 0.7
Physical Health (1–10)	7.4 ± 0.4	8.2 ± 0.2	8.0 ± 0.5	8.3 ± 0.5	7.5 ± 0.5	7.8 ± 0.4
Satiety (1–10) ^†^	8.2 ± 0.4	7.3 ± 0.4	8.0 ± 0.5	6.4 ± 0.6	8.5 ± 0.5	6.9 ± 0.8

Values are mean ± SEM. ^†^: Time effect for satiety (*p* = 0.005). No other statistically significant differences noted (*p* > 0.05).

### 3.2. Hemodynamic and Anthropometric Data

No time or interaction effects were noted for any hemodynamic or anthropometric variable (*p* > 0.05). However, group effects were noted for the following variables: age (*p* = 0.05), Modified DF < Traditional DF; total body fat (*p* = 0.04), Modified DF < Traditional DF and Unrestricted Vegan; fat free mass (*p* = 0.05), Modified DF > Traditional DF; heart rate (*p* = 0.02), Modified DF < Traditional DF; systolic BP (*p* = 0.01), Modified DF and Traditional DF < Unrestricted Vegan. A trend for a time effect for systolic BP was also noted (*p* = 0.06), with values decreasing across time (*i.e.*, pre- to post-intervention). An approximate 2 kg weight loss was observed in subjects assigned to the traditional DF, with an approximate 1 kg weight loss noted in subjects assigned to the modified DF (pre- to post-intervention). Systolic BP was reduced approximately 7 mmHg in subjects assigned to the traditional DF, with an approximate 5 mmHg reduction noted in subjects assigned to the modified DF and the unrestricted vegan plan (pre- to post-intervention). Data are presented in Table 2.

**Table 2:** Subject characteristics before and after a 21-day period of dietary modification.

Variable	Traditional DF Pre	Traditional DF Post	Modified DF Pre	Modified DF Post	Unrestricted Vegan Pre	Unrestricted Vegan Post
Age (years) *	38.1 ± 4.7	NA	27.9 ± 3.8	NA	31.5 ± 4.5	NA
Height (cm)	165.1 ± 2.8	NA	167.7 ± 2.3	NA	168.9 ± 2.4	NA
Weight (kg)	69.4 ± 3.4	67.4 ± 3.3	72.4 ± 5.0	71.4 ± 5.2	75.0 ± 4.6	74.4 ± 4.6
BMI (kg∙m^−2^)	25.6 ± 1.4	24.8 ± 1.3	25.8 ± 1.8	25.4 ± 1.9	26.2 ± 1.3	26.0 ± 1.3
Waist (cm)	78.5 ± 3.6	78.6 ± 3.6	80.3 ± 4.0	78.9 ± 4.2	82.3 ± 4.4	82.5 ± 4.0
Hip (cm)	103.5 ± 2.9	103.8 ± 2.3	103.3 ± 3.0	102.3 ± 3.1	101.3 ± 3.4	103.0 ± 2.4
Waist:Hip	0.76 ± 0.02	0.76 ± 0.02	0.78 ± 0.02	0.77 ± 0.02	0.81 ± 0.03	0.80 ± 0.03
Total Body Fat (%) *	31.1 ± 2.5	30.2 ± 2.4	28.2 ± 2.9	27.8 ± 3.0	33.1 ± 2.4	32.4 ± 2.3
Trunk Body Fat (%)	31.1 ± 2.5	29.1 ± 1.8	26.3 ± 2.6	25.2 ± 2.9	30.5 ± 2.9	29.2 ± 2.8
Fat Mass (kg)	21.8 ± 2.4	20.4 ± 2.1	21.0 ± 3.5	20.5 ± 3.8	25.3 ± 2.8	24.6 ± 2.7
Fat Free Mass (kg) *	47.6 ± 2.7	47.0 ± 2.9	51.4 ± 3.1	50.9 ± 3.1	49.7 ± 2.7	49.8 ± 2.8
Heart Rate (bpm) *	80.2 ± 4.2	78.5 ± 4.0	69.1 ± 2.9	69.9 ± 3.8	73.2 ± 3.5	76.0 ± 4.0
Systolic BP (mmHg) *^,†^	117.3 ± 4.4	110.6 ± 2.3	116.8 ± 2.4	111.5 ± 3.3	125.2 ± 3.3	120.6 ± 3.8
Diastolic BP (mmHg)	73.2 ± 3.0	71.1 ± 3.0	70.2 ± 3.0	68.6 ± 2.9	67.3 ± 4.2	74.0 ± 4.2

Values are mean ± SEM. *: Group effect for age (*p* = 0.05); Modified DF < Traditional DF. *: Group effect for total body fat (*p* = 0.04); Modified DF < Traditional DF and Vegan. *: Group effect for fat free mass (*p* = 0.05); Modified DF > Traditional DF. *: Group effect for heart rate (*p* = 0.02); Modified DF < Traditional DF. *: Group effect for systolic BP (*p* = 0.01); Modified DF and Traditional DF < Vegan. ^†^: Trend for time effect for systolic BP (*p* = 0.06). No other statistically significant differences noted (*p* > 0.05).

### 3.3. Biochemical Data

With regards to the complete blood count and metabolic panel, no time or interaction effects were noted (*p* > 0.05; data not shown). An approximate 33% reduction was noted from pre- to post-intervention for insulin in subjects assigned to the traditional DF. With regards to the lipid panel and oxidative stress data, no group, time, or interaction effects were noted (*p* > 0.05). However, trends were noted for the following variables: time effect for cholesterol (*p* = 0.10); group effect for triglycerides (*p* = 0.07), Traditional DF < Unrestricted Vegan; group effect for VLDL (*p* = 0.07), Traditional DF < Unrestricted Vegan. An approximate 20% reduction was noted in total and LDL cholesterol for subjects in the traditional DF group, while this decrease was approximately 10% for subjects in the modified DF group (from pre- to post-intervention). No decrease in total or LDL cholesterol for noted for subjects in the unrestricted vegan group from pre- to post-intervention. Biochemical data are presented in Table 3.

### 3.4. Dietary Data

As might be anticipated based on the research design, many differences were noted in dietary intake between groups and across time (*i.e.*, from the week prior to beginning the dietary plan (pre-intervention) to the final week of the plan (post-intervention)). These included: kilocalories (group effect: *p* = 0.04, Modified DF > Traditional DF; time effect: *p* = 0.0008), protein grams (group effect: *p* < 0.0001, Traditional DF and Unrestricted Vegan < Modified DF; time effect: *p* = 0.004; interaction effect: *p* = 0.03), protein percent (group effect: *p* < 0.0001, Traditional DF and Unrestricted Vegan < Modified DF; interaction effect: *p* < 0.0001), carbohydrate percent (time effect: *p* < 0.0001; interaction effect: *p* = 0.05), fiber (time effect: *p* = 0.0004), sugar (group effect: *p* = 0.02, Modified DF > Unrestricted Vegan), fat grams, fat percent, saturated fat grams, trans fat grams (time effect: *p* < 0.0001), cholesterol (group effect: *p* = 0.004, Modified DF > Traditional DF and Unrestricted Vegan; time effect: *p* < 0.0001; interaction effect: *p* = 0.02), vitamin C (group effect: *p* = 0.004, Modified DF > Unrestricted Vegan; time effect: *p* = 0.006), vitamin E (group effect: *p* = 0.05, Modified DF > Unrestricted Vegan), vitamin A (time effect: *p* = 0.01), selenium (group effect: *p* = 0.005). No other effects of statistical significance were noted (*p* > 0.05). Data are presented in Table 4.

**Table 3:** Biochemical data of subjects before and after a 21-day period of dietary modification.

Variable	Traditional DF Pre	Traditional DF Post	Modified DF Pre	Modified DF Post	Unrestricted Vegan Pre	Unrestricted Vegan Post
C-Reactive Protein (mg·L^−1^)	1.6 ± 0.5	1.8 ± 0.7	1.9 ± 0.6	1.5 ± 0.4	1.8 ± 0.3	1.9 ± 0.7
Insulin (µU·mL^−1^)	10.1 ± 2.5	6.7 ± 1.2	9.1 ± 1.5	9.8 ± 2.2	8.1 ± 1.0	8.5 ± 1.4
HOMA-IR	2.2 ± 0.6	1.5 ± 0.3	1.9 ± 0.3	2.3 ± 0.6	1.8 ± 0.3	1.9 ± 0.4
Glucose (mg·dL^−1^)	86.4 ± 4.0	86.9 ± 2.2	87.3 ± 2.7	92.1 ± 2.2	88.7 ± 3.8	86.3 ± 4.5
Cholesterol (mg·dL^−1^) ^†^	179.2 ± 8.7	146.5 ± 7.1	187.8 ± 13.1	169.3 ± 13.7	165.5 ± 12.4	168.3 ± 13.5
HDL-C (mg·dL^−1^)	64.9 ± 3.5	55.9 ± 2.7	68.9 ± 5.5	61.0 ± 5.0	64.9 ± 5.5	61.5 ± 4.0
VLDL-C (mg·dL^−1^) *	13.0 ± 1.4	12.2 ± 1.8	16.2 ± 2.0	15.5 ± 1.7	17.8 ± 3.3	17.6 ± 3.1
LDL-C (mg·dL^−1^)	101.3 ± 6.8	78.4 ± 5.2	102.7 ± 9.2	92.8 ± 9.6	82.8 ± 10.8	89.2 ± 11.0
Total:HDL-C	2.8 ± 0.1	2.6 ± 0.1	2.8 ± 0.2	2.8 ± 0.1	2.7 ± 0.3	2.8 ± 0.3
Nitrate/Nitrite (µmol·L^−1^)	22.3 ± 5.2	23.9 ± 4.7	23.7 ± 8.2	33.6 ± 9.7	25.2 ± 5.7	39.0 ± 12.9
Malondialdehyde (µmol·L^−1^)	0.8 ± 0.1	0.6 ± 0.1	0.7 ± 0.1	0.7 ± 0.1	0.8 ± 0.2	0.7 ± 0.1
Advanced Oxidation Protein Products (µmol·L^−1^)	65.9 ± 5.6	61.6 ± 7.5	70.2 ± 9.7	70.0 ± 13.3	61.4 ± 7.3	61.1 ± 5.1

Values are mean ± SEM. ^†^: Trend for time effect for cholesterol (*p* = 0.10). *: Trend for group effect for triglycerides (*p* = 0.07); Traditional DF < Vegan. *: Trend for group effect for VLDL (*p* = 0.07); Traditional DF < Vegan. No other statistically significant differences noted (*p* > 0.05).

**Table 4:** Dietary data of subjects before and during the final seven days of a 21-day period of dietary modification.

Variable	Traditional DF Pre	Traditional DF Post	Modified DF Pre	Modified DF Post	Unrestricted Vegan Pre	Unrestricted Vegan Post
Kilocalories *^,†^	1753 ± 133	1147 ± 93	2049 ± 159	1636 ± 253	1743 ± 122	1335 ± 118
Protein (g) *^,†,‡^	68 ± 6	34 ± 4	82 ± 6	86 ± 13	69 ± 6	40 ± 6
Protein (%) *^,‡^	15 ± 1	12 ± 1	16 ± 1	22 ± 1	16 ± 1	11 ± 1
Carbohydrate (g)	218 ± 17	189 ± 15	259 ± 23	226 ± 34	218 ± 15	207 ± 14
Carbohydrate (%) ^†,‡^	49 ± 1	67 ± 2	50 ± 1	56 ± 3	50 ± 3	64 ± 3
Fiber (g) ^†^	16 ± 2	29 ± 3	19 ± 3	29 ± 5	15 ± 2	20 ± 2
Sugar (g) *	82 ± 9	65 ± 9	97 ± 11	95 ± 11	64 ± 10	68 ± 6
Fat (g) ^†^	66 ± 6	32 ± 4	74 ± 7	47 ± 11	68 ± 9	40 ± 6
Fat (%) ^†^	34 ± 1	25 ± 2	32 ± 1	24 ± 2	34 ± 2	26 ± 3
Saturated Fat (g) ^†^	20 ± 2	4 ± 1	24 ± 2	9 ± 2	21 ± 3	10 ± 2
Monounsaturated Fat (g)	11 ± 3	7 ± 2	12 ± 2	16 ± 6	9 ± 1	5 ± 1
Polyunsaturated Fat (g)	6 ± 1	5 ± 0	6 ± 1	8 ± 2	7 ± 1	4 ± 1
Trans Fat (g) ^†^	0.7 ± 0	0.0 ± 0	1.0 ± 0	0.2 ± 0	0.8 ± 0	0.4 ± 0
Omega 3 (mg)	0.4 ± 0	0.5 ± 0	0.6 ± 0	0.8 ± 0	0.6 ± 0	0.3 ± 0
Omega 6 (mg)	4 ± 1	3 ± 1	4 ± 1	6 ± 2	4 ± 1	3 ± 1
Cholesterol (mg) *^,†,‡^	240 ± 41	3 ± 2	236 ± 26	153 ± 33	171 ± 27	43 ± 18
Vitamin C (mg) *^,†^	59 ± 11	110 ± 12	87 ± 23	143 ± 25	41 ± 8	61 ± 17
Vitamin E (mg) *	4 ± 1	5 ± 2	4 ± 1	7 ± 2	3 ± 0	2 ± 1
Vitamin A (RE) ^†^	263 ± 50	467 ± 89	263 ± 45	481 ± 123	231 ± 52	350 ± 90
Selenium (µg) *	31 ± 7	14 ± 3	49 ± 8	60 ± 17	38 ± 6	32 ± 6

Values are mean ± SEM. *: Group effect for kilocalories (*p* = 0.04); Modified DF > Traditional DF. ^†^: Time effect for kilocalories (*p* = 0.0008). *: Group effect for protein grams (*p* < 0.0001); Traditional DF and vegan < Modified DF. ^†^: Time effect for protein grams (*p* = 0.004). ^‡^: Interaction effect for protein grams (*p* = 0.03). *: Group effect for protein percent (*p* < 0.0001); Traditional DF and vegan < Modified DF. ^‡^: Interaction effect for protein percent (*p* < 0.0001). ^†^: Time effect for carbohydrate percent (*p* < 0.0001). ^‡^: Interaction effect for carbohydrate percent (*p* = 0.05). ^†^: Time effect for fiber (*p* = 0.0004). * Group effect for sugar (*p* = 0.02); Modified DF > vegan. ^†^: Time effect for fat grams, fat percent, saturated fat grams, trans fat grams (*p* < 0.0001). *: Group effect for cholesterol (*p* = 0.004); Modified DF > Traditional DF and vegan. ^†^: Time effect for cholesterol (*p* < 0.0001). ^‡^: Interaction effect for cholesterol (*p* = 0.02). *: Group effect for vitamin C (*p* = 0.004); Modified DF > vegan. ^†^: Time effect for vitamin C (*p* = 0.006). *: Group effect for vitamin E (*p* = 0.05); Modified DF > vegan. ^†^: Time effect for vitamin A (*p* = 0.01). *: Group effect for selenium (*p* = 0.005). No other statistically significant differences noted (*p* > 0.05).

## 4. Discussion

Our findings indicate that all three diet plans may have benefits in terms of enhancing selected outcomes related to human health. Both a traditional and modified DF may improve blood pressure and blood lipids in a clinically meaningful manner if such changes can be sustained over a longer period of time. A traditional DF also results in a relevant reduction in blood insulin and oxidative stress biomarkers. However, additional longer-term studies are needed to provide more information pertaining to the health benefits of the Daniel Fast plans. It should also be noted that the study sample size is relatively small and future studies inclusive of a larger sample are needed to extend these findings.

The data presented in this study are in reference to a wide range of relatively healthy individuals, predominantly women, all but two (both in the unrestricted vegan group) of whom claimed to regularly perform exercise (traditional DF = 5.6 h/week; modified DF = 4.6 h/week; unrestricted vegan = 4.4 h/week). Moreover, the results were obtained following just 21 days of adherence to the prescribed diet plan. As indicated above, longer-term studies are needed to extend these findings.

As in our prior studies with the DF, self-reported compliance to the dietary plans was excellent, approaching 100% for all groups. While the plan was only three weeks in duration, the high compliance rate highlights the fact that these diet plans may be reasonably maintained by committed individuals. Indeed, our recent work involving a six month period of both a traditional and modified DF confirms this [13]; although mean kilocalorie intake was closer to 1400–1500 per day for the traditional and modified DF respectively, which appears more realistic than the ~1150 figure noted in the present study for the traditional DF. Additional longer-term studies focused on dietary compliance are needed to determine if individuals can follow these plans for extended periods of time—at lengths that are likely necessary to foster meaningful health benefits related to longevity and disease. It should also be noted that since compliance was self-reported, the possibility exists that participants may have overestimated their adherence to the plans. For example, while the unrestricted vegan group reported 94% compliance, the diet analysis indicated a mean post-intervention cholesterol intake of 43 mg. Although this is a significant drop from their pre-intervention mean value of 171 mg, if strictly adhering to the plan, we would expect that cholesterol intake would be even lower.

Although not of statistical significance, subjects assigned to the traditional DF group reported an approximate 10% increase in perceived mental and physical health. Systolic BP was also reduced approximately 7 mmHg in subjects assigned to the traditional DF, with an approximate 5 mmHg reduction noted in subjects assigned to the modified DF and the unrestricted vegan plan. The reduction in BP with the plant-based diets has been discussed previously [14] and may be related to multiple components within the diet including the quantity and type of dietary fat, the intake of fruit and vegetables, the amount of dietary protein, and the overall kilocalorie intake [15].

Regarding biochemical outcomes, blood lipids were reduced with the DF plans and to a greater extent with the traditional plan. In our prior study comparing the two plans, results were relatively similar between plans [6]. From a clinical perspective, both diet plans may be viewed as health-enhancing with regards to blood lipids. Interestingly, both total and LDL cholesterol were increased in subjects following the unrestricted vegan plan; this despite a lower dietary cholesterol intake in the unrestricted vegan group compared to the modified DF group. It is possible that the fatty acid composition of the diet (saturated, monounsaturated, polyunsaturated fats), as well as the fiber intake, could be responsible for the differences between groups.

Aside from blood lipids, insulin was reduced in the traditional DF group, while malondialdehyde also was decreased. We have noted the same in our prior studies using the traditional DF [8,9]. No changes in these measures were noted in either the modified DF or unrestricted vegan groups. It is possible that the greater reduction in kilocalorie intake for those in the traditional DF group (Table 4) was responsible for the noted differences, as a reduction in dietary energy is known to improve redox state [16] and may have implications for improved insulin sensitivity [17]. For example, subjects assigned to the traditional DF reduced kilocalories by approximately 35%, while those in the modified DF and unrestricted vegan plans only experienced a reduction of approximately 20% and 23%, respectively. It is likely that a larger sample of subjects is needed to better explain the impact of these dietary plans on metabolic and oxidative stress biomarkers. Future studies should aim to enroll a higher number of participants and possibly monitor them over a longer time course.

While multiple dietary variables were measured and noted to be different between diet groups and across time, it is unknown which variable(s) most contributed to the effects observed. That said, it is likely that the decrease in total kilocalorie intake may be the most important variable driving the outcomes, with consideration for dietary fiber, cholesterol, and fat also being responsible for our findings—in particular in relation to blood lipids. Of course, variables such as carbohydrate type and various additives and preservatives that may have been contained within foods consumed by those assigned to the unrestricted vegan diet may have impacted the variables measured in the present study. For example, the kilocalorie intake was lower for the unrestricted vegan group as compared to the modified DF group at the post-intervention time. However, the overall results were more favorable for the modified DF group. Moreover, data obtained from a recent animal study in our lab indicates that despite receiving a similar daily food ration (kcal intake), animals benefit much more from consuming a diet formulated to be similar to the traditional DF, as compared to a typical American diet consisting of high amounts of saturated fat and simple sugar [18].

It should be noted that mean kilocalorie intake was quite low at the post-intervention time for the traditional DF group. Many subjects reported a significant increase in satiety when following a traditional DF plan, leading to a reduction in energy intake. This may have been the case in the present study, in addition to the possibility of under-reporting in dietary intake.

## 5. Conclusions

The data from the present study indicate that otherwise healthy men and women including normal weight, overweight, and obese individuals can improve certain measures of health by adopting a traditional or modified DF dietary plan. All diet plans were well-tolerated by subjects, and many subjects who followed the DF plans noted an increase in nutritional knowledge by following the plan for a mere three weeks (e.g., understanding of food labels, recognizing what is contained within commonly consumed foods). Follow-up studies should include a larger sample size and an extended time frame of dietary adherence in an attempt to determine both the feasibility and potential health benefits of these plans in both men and women. Studies may also be conducted using individuals with known cardiovascular or metabolic disease, as clinically relevant results may be observed following these dietary plans.

## References

[1] Holloszy J.O., Fontana L. (2007). Caloric restriction in humans. Exp. Gerontol..

[2] Koubova J., Guarente L. (2003). How does calorie restriction work?. Genes. Dev..

[3] Spindler S.R. (2009). Caloric restriction: From soup to nuts. Ageing Res. Rev..

[4] Everitt A.V., Hilmer S.N., Brand-Miller J.C., Jamieson H.A., Truswell A.S., Sharma A.P., Mason R.S., Morris B.J., le Couteur D.G. (2006). Dietary approaches that delay age-related diseases. Clin. Interv. Aging.

[5] Simpson S.J., Raubenheimer D. (2009). Macronutrient balance and lifespan. Aging (Albany NY).

[6] Alleman R.J., Harvey I.C., Farney T.M., Bloomer R.J. (2013). Both a traditional and modified Daniel, Fast improve the cardio-metabolic profile in men and women. Lipids Health Dis..

[7] Bloomer R.J., Trepanowski J.F., Kabir M.M., Alleman R.J., Dessoulavy M.E. (2012). Impact of short-term dietary modification on postprandial oxidative stress. Nutr. J..

[8] Bloomer R.J., Kabir M.M., Trepanowski J.F., Canale R.E., Farney T.M. (2011). A 21 day Daniel Fast improves selected biomarkers of antioxidant status and oxidative stress in men and women. Nutr. Metab. (Lond).

[9] Bloomer R.J., Kabir M.M., Canale R.E., Trepanowski J.F., Marshall K.E., Farney T.M., Hammond K.G. (2010). Effect of a 21 day Daniel Fast on metabolic and cardiovascular disease risk factors in men and women. Lipids Health Dis..

[10] Trepanowski J.F., Kabir M.M., Alleman R.J., Bloomer R.J. (2012). A 21-day Daniel fast with or without krill oil supplementation improves anthropometric parameters and the cardiometabolic profile in men and women. Nutr Metab (Lond).

[11] Dwyer J.T. (1988). Health aspects of vegetarian diets. Am. J. Clin. Nutr..

[12] Key T.J., Appleby P.N., Rosell M.S. (2006). Health effects of vegetarian and vegan diets. Proc. Nutr. Soc..

[13] Bloomer R.J., Toline A.H. (2014). Participant compliance to a 6-month traditional and modified Daniel Fast. J. Fasting Health.

[14] Yokoyama Y., Nishimura K., Barnard N.D., Takegami M., Watanabe M., Sekikawa A., Okamura T., Miyamoto Y. (2014). Vegetarian diets and blood pressure: A meta-analysis. JAMA Intern. Med..

[15] Beilin L.J., Burke V. (1995). Vegetarian diet components, protein and blood pressure: Which nutrients are important?. Clin. Exp. Pharmacol. Physiol..

[16] Kowaltowski A.J. (2011). Caloric restriction and redox state: Does this diet increase or decrease oxidant production?. Redox Rep..

[17] Soare A., Weiss E.P., Pozzilli P. (2014). Benefits of caloric restriction for cardiometabolic health, including type 2 diabetes mellitus risk. Diabetes Metab. Res. Rev..

[18] Bloomer R.J. (2015). Comparison of a vegan and a Western Diet plan on health specific measures in Long-Evans rats.

